# ﻿Genetic diversity among sea snakes of the genus *Hydrophis* (Elapidae, Reptilia) in the Persian Gulf and Gulf of Oman

**DOI:** 10.3897/zookeys.1158.101347

**Published:** 2023-04-20

**Authors:** Seyyed Saeed Hosseinian Yousefkhani, Amaal Yasser, Murtada Naser, Mohsen Rezaie-Atagholipour, Majid Askari Hesni, Fariba Yousefabadi, Eskandar Rastegar Pouyani

**Affiliations:** 1 Department of Animal Science, School of Biology, Damghan University, Damghan, Iran; 2 Marine Science Centre, University of Basrah, Basra, Iraq; 3 Australian Rivers Institute, Griffith University, 170 Kessels Road, Nathan, Queensland 4111, Australia; 4 School of Environment and Science, Griffith University, 170 Kessels Road, Nathan, Queensland, 4111, Australia; 5 Qeshm Environmental Conservation Institute (QECI), Qeshm Island, Hormozgan Province, Iran; 6 Department of Biology, Faculty of Sciences, Shahid Bahonar University of Kerman, Kerman Province, Iran; 7 Department of Biology, Faculty of Science, Ferdowsi University of Mashhad, Mashhad, Iran; 8 Department of Biology, Hakim Sabzevari University, Sabzevar, Iran

**Keywords:** Dispersal, Indian Ocean, microhabitat adaptation, true sea snakes, variation

## Abstract

Sea snakes of the genus *Hydrophis* are important components of animal diversity in Iranian waters of the Persian Gulf and Gulf of Oman. Ten species of *Hydrophis* have been identified from the these waters and, in this study, genetic structure of seven species was compared with other populations in the eastern Indian Ocean and the West Pacific. We found that six species (*H.platurus*, *H.cyanocinctus*, *H.spiralis*, *H.schistosus*, *H.gracilis*, and *H.lapemiodes*) show high genetic similarity with conspecific populations in the Indian Ocean and Australia. However, *H.curtus* from southern Iran shows a high level of genetic differentiation from conspecific populations in Sri Lanka and Indonesia (0.6% and 6% genetic distance from Sri Lankan samples for *16S* and *COI* gene fragments, respectively). Variation between Iranian and Southeast Asian populations may reflect new genetic lineages and suggest the need of further morphological evaluations to re-evaluate their taxonomic position.

## ﻿Introduction

Sea snakes are a well-known part of marine ecosystems and play an irreplaceable role in the food chain as both predators of small marine fauna and prey for larger predators within their geographical distribution range, which comprises the tropical Indo-Pacific (Voris 1972; Udyawer et al. 2018). True sea snakes of the tribe Hydrophiini share a common ancestor approximately six million years ago, but the major speciation events and radiation within the group and the genus *Hydrophis* have taken place over the last 3.5 million years (Sanders et al. 2013). While studies on different subjects (e.g., Ukuwela et al. 2012, 2016) have been conducted on sea snakes on the eastern half of their geographic range (i.e., Southeast Asia, India, Indonesia, and northern Australia), their populations remain relatively understudied in the western Indian Ocean (Rezaie-Atagholipour et al. 2016).

Iranian coastal waters of the Persian Gulf and Gulf of Oman are known for their high biological diversity (Owfi et al. 2016) and share a marine herpetofauna with the Indian Ocean (Gasperetti 1988; Heatwole 1999). Ten species of *Hydrophis* have been recorded in these gulfs: *H.platurus*, *H.schistosus*, *H.curtus*, *H.viperinus*, *H.spiralis*, *H.cyanocinctus*, *H.ornatus*, *H.lapemiodes*, *H.gracilis*, and *H.cantoris* (Rezaie-Atagholipour et al. 2016; Rajabizadeh 2019). Among these, *H.platurus* has a wider geographic range toward eastern Africa (Heatwole 1999), but the westernmost ranges of the other species extend to the Persian Gulf (Rezaie-Atagholipour et al. 2016). The semi-enclosed basin of the Persian Gulf (i.e., connected to the Gulf of Oman and western Indian Ocean via the narrow Strait of Hormuz) and its unique extreme environment with high salinity and temperatures (i.e., sea surface temperatures can exceed 34 °C and salinity more than 39 ppt in most areas; Sheppard et al. 2010) may pose ecological and geographical barriers and limit gene flow between the Gulf and Indian Ocean marine communities (e.g., Natoli et al. 2017). Therefore, it seems important to compare the genetic structure of sea snakes, which are known to have undergone rapid speciation (see Sanders et al. 2013), between the Persian Gulf and the rest of the Indo-Pacific.

In this study, available genetic markers (16S rRNA, *COI*, and *G1888*) of species of sea snakes of the genus *Hydrophis* from the Persian Gulf and the Gulf of Oman were compared to populations in other areas of the Indian Ocean, Southeast Asia, and Indonesia. Examining the genetic structure of the species in the Persian Gulf and comparing them with other populations can illustrate the degree of genetic connection between these populations.

## ﻿Materials and methods

### ﻿Tissue sampling and DNA extraction

**Figure 1.**
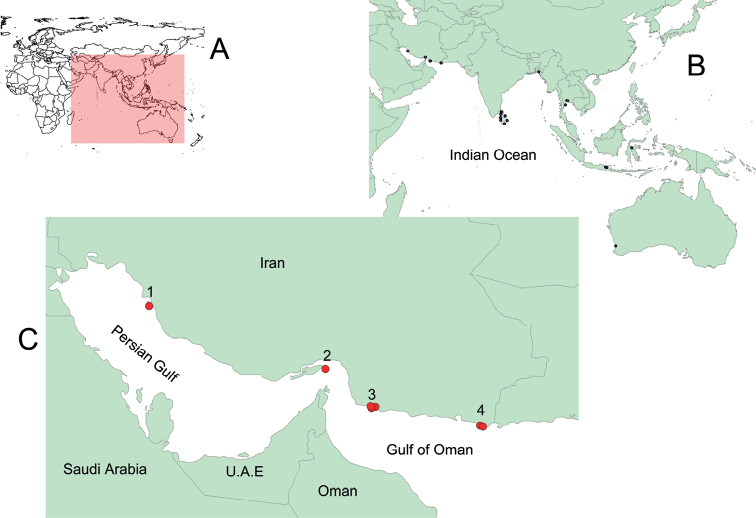
**A** map of the world and the selected region as study area **B** localities in South and Southeast Asia from which samples were used in this study **C** localities in southern Iran where samples were collected: 1 = Bushehr; 2 = Larak Island; 3 = Jask; 4 = Beris and Pasabandar.

Tissue samples were obtained from the specimens collected in the southern coastal regions of Iran (Persian Gulf and Gulf of Oman) during fieldwork in 2013 (Rezaie-Atagholipour et al. 2016) and were preserved in 96% ethanol. A total of 31 samples used in this study belong to seven species from four localities in southern Iran (Fig. 1, Suppl. material 1) and 27 comparative sequences were downloaded from GenBank. Sequences of *Ephalophisgreyae*, used as the outgroup, were also downloaded from GenBank (Suppl. material 1). Total genomic DNA was extracted from muscle tissue using the standard proteinase K – salt method (Kabir et al. 2006), and the quality and concentration of extracted DNA were measured using Nanodrop 1000.

### ﻿Mitochondrial and nuclear fragment sequences

Two mitochondrial sequences, 16S rRNA (*16S*) (Kocher et al. 1989) and cytochrome oxidase subunit 1 (*COI*), and one anonymous nuclear marker (*G1888*) (Bertozzi et al. 2012) were used to reconstruct the molecular phylogenetic relationships between Iranian and Southeast Asian species of sea snakes. Two of these markers were obtained from previous successful studies (Lukoschek and Keogh 2006; Sanders et al. 2013), but *COI* is a new marker generated for sea snakes in the present study. The following primers were used for each gene fragment: *16S*: 16SL 5'-CGCCTGTTT ATCAAAAACAT-3'/ 16SH 5'-CCGGTCTGAACTCAGATCACG-3'; *COI*: RepCOIF 5'-TNTTMTCAACNAACCACAAAGA-3'/ RepCOIR 5'-ACTTCTGGRTGKCCAAARAATCA-3'; G1888: G1888F 5'- CAGGGCCTTGCCTTGTGCCA-3'/ G1888R 5'-ACCTCTGCGCACTATGACTCTTGA-3' (Bertozzi et al. 2012). All DNA sequences were amplified using a standard PCR protocol with denaturation at 94 °C for 5 min, annealing temperatures of 49 °C for mitochondrial fragments and 52 °C for anonymous nuclear marker for 45 s, and extension at 72 °C for 70 s (36 cycles), and the final extension for 8 min. Sequencing of the PCR products was performed by the Kodon Genetic Group in Tehran, Iran. Sequences of the same species and the same genetic fragments from Southeast Asia and the outgroup were downloaded from GenBank and added to the dataset (Suppl. material 1). All sequences were aligned using the ClustalW algorithm implemented within Bioedit v. 7.0.9.0 (Hall 1999), and the protein coding genes were translated to amino acid sequences using MEGA v. 6.0 (Tamura et al. 2013) to check for internal stop codons and to determine the correct reading frame. The sequences generated in this study were deposited in GenBank and will be added into Suppl. material 1. Uncorrected genetic distances (*p*-distances) were calculated (for *16S* and *COI*) and number of variable sites (*V*) and parsimony informative sites (*Pi*) were obtained using MEGA v. 6.0 for all markers. The sequences of the *COI* gene fragment were few and we only calculate genetic distance among some clades that revealed relatively high values.

### ﻿Molecular phylogenetic analyses and haplotype network

The one mitochondrial and the nuclear fragment were concatenated (total length: 1015 bp; *16S*: 523 bp; *G1888*: 492 bp) and *COI* gene fragment (708 bp) separately were considered to reconstruction phylogenetic trees using Bayesian inference (BI) and maximum-likelihood (ML) methods. The best substitution models for each alignment were: TrNef+I, F81+I, TIM+G, GTR+I+G and TVM+I+G for the first, second, and third codon positions of *COI* and *16S* and *G1888*, respectively. RaxML v. 7.4.2 (Stamatakis 2006) as implemented in RaxmlGUI v. 1.3 (Silvestro and Michalak 2012) was employed for the ML analyses. The substitution model was set to GTR+G+I in RaxMl software for the molecular phylogenetic reconstruction. Bootstrapping was set to 1000 replicates to estimate nodal support, and the analysis was run as a heuristic search (Felsenstein 1985). MrBayes v. 3.2.1 (Ronquist et al. 2012) was used to run the BI analysis and the number of generations were set as 10^7^ with a sample frequency of every 1000 generations, four Markov chains, and a burn-in with each 1000 samples.

The relationships among lineages and subclades of *Hydrophis* were assessed with the mitochondrial *16S rRNA* gene, because the number of sequences of *16S* covered all species. Aligned sequences were entered in DNAsp v. 5.0. (Librado and Rozas 2009) and a *.rdf extension file was created. The haplotype network was then constructed using the median-joining method in Network 1.2.1.

## ﻿Results

**Figure 2.**
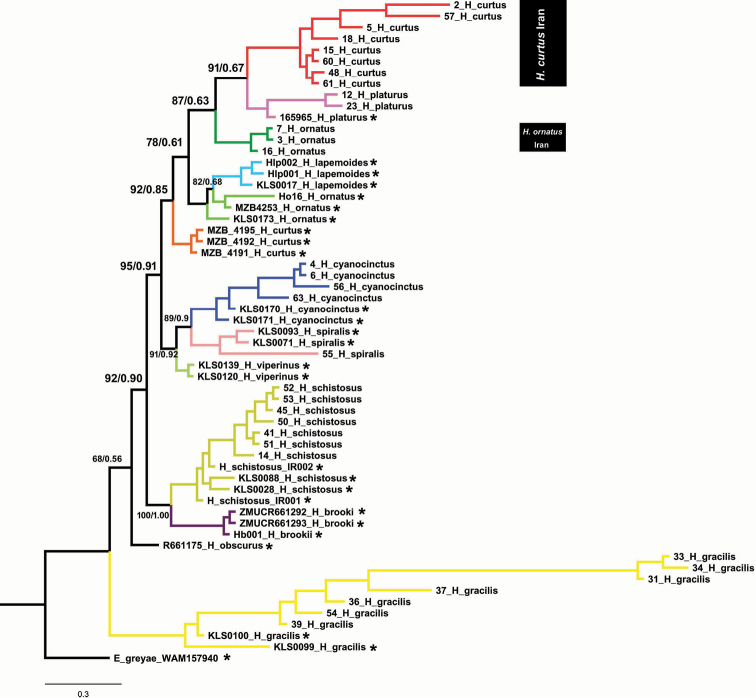
Molecular phylogenetic tree of the genus *Hydrophis*. Red and green clades indicate distinct populations of *H.curtus* and *H.ornatus* from the Persian Gulf relative to other populations in the Indian Ocean. Data from samples with an asterisk (*) were downloaded from GenBank.

Our dataset includes 59 samples containing two mitochondrial gene fragments *16S* (495 bp; 77 *V*; 40 *Pi*) and *COI* (661 bp; 158 *V*; 112 *Pi*) and one nuclear anonymous fragment G1888 (375 bp; 265 *V*; 250 *Pi*) totaling 1531 bp. Both the ML and BI trees show similar topology and therefore we present only the BI tree (Fig. 2). Our results show that all species of *Hydrophis* in the Persian Gulf and Gulf of Oman similar with their corresponding species in Southeast Asia except *H.curtus* and *H.lapemiodes*. *Hydrophiscurtus* in the western part of its geographic range (Persian Gulf and Gulf of Oman) shows clear divergence from Indian Ocean (Sri Lanka) and Indonesian samples (Ukuwela et al. 2014). *Hydrophisornatus* also shows minor variation between Iranian and Indian Ocean populations, but not as extensive.

**Figure 3.**
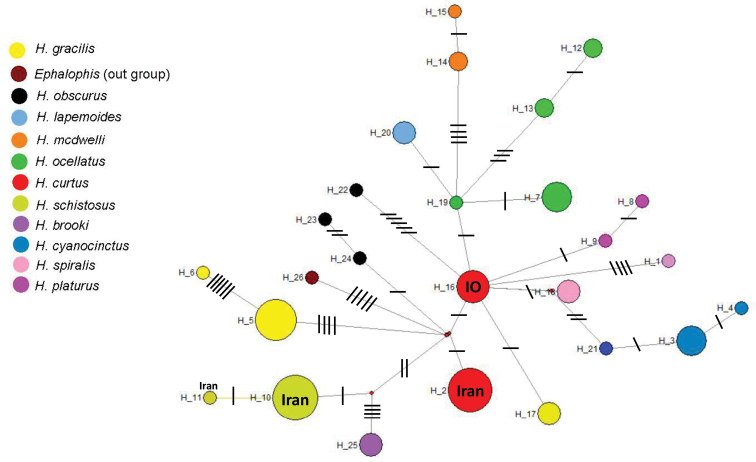
Haplotype network of the genus *Hydrophis* for the *16S* gene fragment. The haplotype colors correspond with the those in the molecular phylogenetic tree in Fig. 2.

Tree topology was used to group the sequences and calculate the uncorrected genetic distance (*p*-distance) among the lineages (Table 1). Mean genetic distance in *16S rRNA* is relatively low (average 3.25% among all lineages) in *Hydrophis*. Iranian lineages of *H.curtus* are differentiated from those in Southeast Asia by 0.6% and 6% in *16S* and *COI* gene fragments, respectively (Tables 1, 2). Two other distinct species, *H.platurus* and *H.viperinus*, show the genetic differentiation of 0.6% in *16S* and 5.1% in *COI* (Tables 1, 2). The haplotype network calculated for the *16S rRNA* fragment confirmed the relationship among lineages in the concatenated phylogenetic tree (Fig. 3). The Iranian clade of *H.curtus* had a separate haplotype (haplotype number 2), but the other conspecific samples (Pacific Ocean) had a different distinct haplotype (haplotype number 16).

**Table 1.** *P*-distances among lineages of sea snakes in *16S* gene fragment: 1 = *H.spiralis*; 2 = *H.curtus* Iran; 3 = *H.cyanocinctus*; 4 = *H.gracilis*; 5 = *H.ornatus*_Iran; 6 = *H.platurus*; 7 = *H.schistosus*; 8 = *H.curtus*; 9 = *H.ornatus*; 10 = *H.viperinus*; 11 = *H.obscurus*; 12 = *H.lapemiodes*; 13 = *H.brooki*.

	1	2	3	4	5	6	7	8	9	10	11	12	13
1													
2	1.8												
3	1.8	1.9											
4	3.6	2.5	3.7										
5	1.7	1.6	1.6	3.4									
6	1.4	1.3	1.3	3.1	0.9								
7	2.3	1.6	2.2	3.4	1.9	1.6							
8	1.2	0.6	1.3	2.5	0.9	0.6	1.6						
9	1.9	1.8	1.8	3.6	0.6	1.2	1.9	1.2					
10	1.4	1.3	1.3	3.1	0.9	0.6	1.6	0.6	1.2				
11	2.6	1.9	2.5	3.1	2.2	1.9	2.2	1.9	2.4	1.9			
12	1.7	1.6	1.6	3.4	0.6	0.9	1.9	0.9	0.8	0.9	2.2		
13	3.6	2.8	3.5	4.6	3.1	2.8	1.9	2.8	3.4	2.8	3.5	3.1	

**Table 2.** *P*-distances among lineages of sea snakes in *COI* gene fragment: 1 = *H.curtus* Iran; 2 = *H.yanocinctus* Iran; 3 = *H.gracilis* Iran; 4 = *H.lapemiodes* Iran; 5 = *H.ornatus* Iran; 6 = *H.platurus* Iran; 7 = *H.schistosus* Iran; 8 = *H.viperinus* Iran; 9 = *H.brooki*; 10 = *H.lapemiodes*; 11 = *H.schistosus*; 12 = *H.cyanocinctus*; 13 = *H.obscurus*; 14 = *H.curtus*; 15 = *H.ornatus*.

	1	2	3	4	5	6	7	8	9	10	11	12	13	14	15
1															
2	6.7														
3	11.2	9.1													
4	7.2	4.7	9.4												
5	5	4.9	10.1	4.7											
6	5.3	6.3	9.8	6	4.3										
7	5.5	5.4	10.4	5.6	4.9	5.8									
8	6.2	5.4	10.4	5.4	3.7	5.5	5.1								
9	8.4	8.3	11.3	7.6	6.9	6.7	8.3	6.9							
10	7	2	9.4	4.9	5.1	6.5	5.6	5.6	8.1						
11	6.1	5.8	11	6.5	5.8	6.1	1.6	5.8	9.2	6					
12	6.2	1.4	9.5	5.1	4.3	5.7	5.8	5.1	8.3	1.6	6.1				
13	5.5	5.4	9.1	5.6	5.1	5.5	5.4	5.4	7.1	5.6	6.3	5.1			
14	6	5.9	10.9	6.4	4.9	5.3	5.4	6.1	7.4	6.1	6.3	5.8	5.6		
15	4.8	4.7	9.9	4.4	0.7	4	4.7	3.4	6.6	4.9	5.5	4.1	4.9	4.7	

## ﻿Discussion

Sea snakes are one of the most interesting reptiles in biogeographic and phylogeographic studies (Ukuwela et al. 2022). A biogeographical study of the genus *Hydrophis* indicated that the diversification of true sea snakes has been the result of sea level changes during the last 2.5 million years (Ukuwela et al. 2016). Our molecular results indicate that the sea snakes in the Persian Gulf and Gulf of Oman share a genetic structure with Indian Ocean and Southeast Asian taxa (Fig. 2). Among them, *H.curtus* has differentiated relatively more than others, so that it may represent a genetically distinct species. Ukuwela et al. (2014) had the same findings for *H.curtus*. Genetic variation among Persian Gulf populations of *H.curtus* and other populations in southeast Asia (0.6% for *16S* and 6% for *COI*) indicate that the former may representative a new genetic lineage in Southwest Asia (Tables 1, 2). This variation among this taxon indicates that the threshold of genetic variation may reveal distinct populations at the species level, although this variation has not yet been established on the basis of morphology (Rezaie-Atagholipour et al. 2016).

A recent study on the genetic structure of *H.curtus* in the Indo-West Pacific revealed the presence of high variation among its populations. The phylogeny indicated that this species was affected by climatic fluctuations in late Pliocene and early Pleistocene (Ukuwela et al. 2014, 2022). Adding the Iranian dataset confirms that *H.curtus* populations in the Persian Gulf in the western part of its range have a close relationship with those in Sri Lanka and exhibit genetic variability greater than those populations in Southeast Asia (Fig. 2). Other species of *Hydrophis* have not yet been studied, but our study indicates that their differentiation requires conducting a comprehensive study. However, the variation among populations of these species implies a pattern of variation in the Indian Ocean and West Pacific regions that may occur either in Sri Lanka or in the Persian Gulf as well (Lambeck 1996; Voris 2000; Lambeck et al. 2002).

Other species of the genus, *H.platurus*, *H.cyanocinctus*, *H.spiralis*, *H.ornatus*, *H.schistosus* and *H.gracilis*, included in the analyses, clearly placed within their conspecific Southeast Asian clades, and this may indicate high gene flow and recent (i.e. 10,000 years) dispersal from their source populations (Fig. 2). We assume that different local adaptations among *H.curtus* and the other six other taxa make them more variable.

The total diversity of the true sea snakes of the genus *Hydrophis* in the Persian Gulf and Gulf of Oman is 10 species (Rezaie-Atagholipour et al. 2016). According to the literature (Ukuwela et al. 2014; Rezaie-Atagholipour et al. 2016; Rajabizadeh 2019), *H.schistosus*, *H.viperinus*, *H.lapemiodes*, *H.cantoris*, *H.gracilis*, *H.spiralis*, and *H.platurus* are abundant in Gulf of Oman as opposed to the Persian Gulf, which means the species prefer to not inhabit the Gulf. But *H.curtus* and *H.ornatus* are mostly distributed in the Persian Gulf and in the Gulf of Oman. Our results revealed the variation between Persian Gulf and Southeast Asia population of *H.curtus* (6% genetic distance in the *COI* gene fragment) and reveals a new distinct genetic lineage. The genetic difference between DNA sequences of *H.curtus* populations in the Persian Gulf and Pacific Ocean is very likely due to genetic drift. In general, biodiversity conservation requires information (demographic and microhabitat information) from local populations. Only then we can encourage conservationists to reassess the status of the species in the Persian Gulf and the Gulf of Oman, because the latest International Union for the Conservation of Nature (IUCN) status of most species is Least Concern and one is Data Deficient (IUCN 2022).

## Supplementary materials

Supplementary material 1 Information of sequences used in this study and those obtained from GenBank Data type occurences, accession numbers Explanation note Information of sequences used in this study and those obtained from GenBank (sequences with an asterisk, *). Accession numbers for the sequences added to the table. Accession numbers in red related to the new sequences of this study. https://binary.pensoft.net/file/840539This dataset is made available under the Open Database License (http://opendatacommons.org/licenses/odbl/1.0/). The Open Database License (ODbL) is a license agreement intended to allow users to freely share, modify, and use this Dataset while maintaining this same freedom for others, provided that the original source and author(s) are credited.Seyyed Saeed Hosseinian Yousefkhani, Amaal Yasser, Murtada Naser, Mohsen Rezaie-Atagholipour, Majid Askari Hesni, Fariba Yousefabadi, Eskandar Rastegar Pouyani

Supplementary material 2 Bayesian tree of COI gene fragment that clearly shows variation in *Hydrophiscurtus* clade Data type figure https://binary.pensoft.net/file/840540This dataset is made available under the Open Database License (http://opendatacommons.org/licenses/odbl/1.0/). The Open Database License (ODbL) is a license agreement intended to allow users to freely share, modify, and use this Dataset while maintaining this same freedom for others, provided that the original source and author(s) are credited.Seyyed Saeed Hosseinian Yousefkhani, Amaal Yasser, Murtada Naser, Mohsen Rezaie-Atagholipour, Majid Askari Hesni, Fariba Yousefabadi, Eskandar Rastegar Pouyani
