# Supplementary figures and images for: ﻿Genetic diversity among sea snakes of the genus Hydrophis (Elapidae, Reptilia) in the Persian Gulf and Gulf of Oman
Source: Zookeys. 2023 Apr 20;1158:121–31. doi: 10.3897/zookeys.1158.101347 (PMC10193271; doi:10.3897/zookeys.1158.101347)

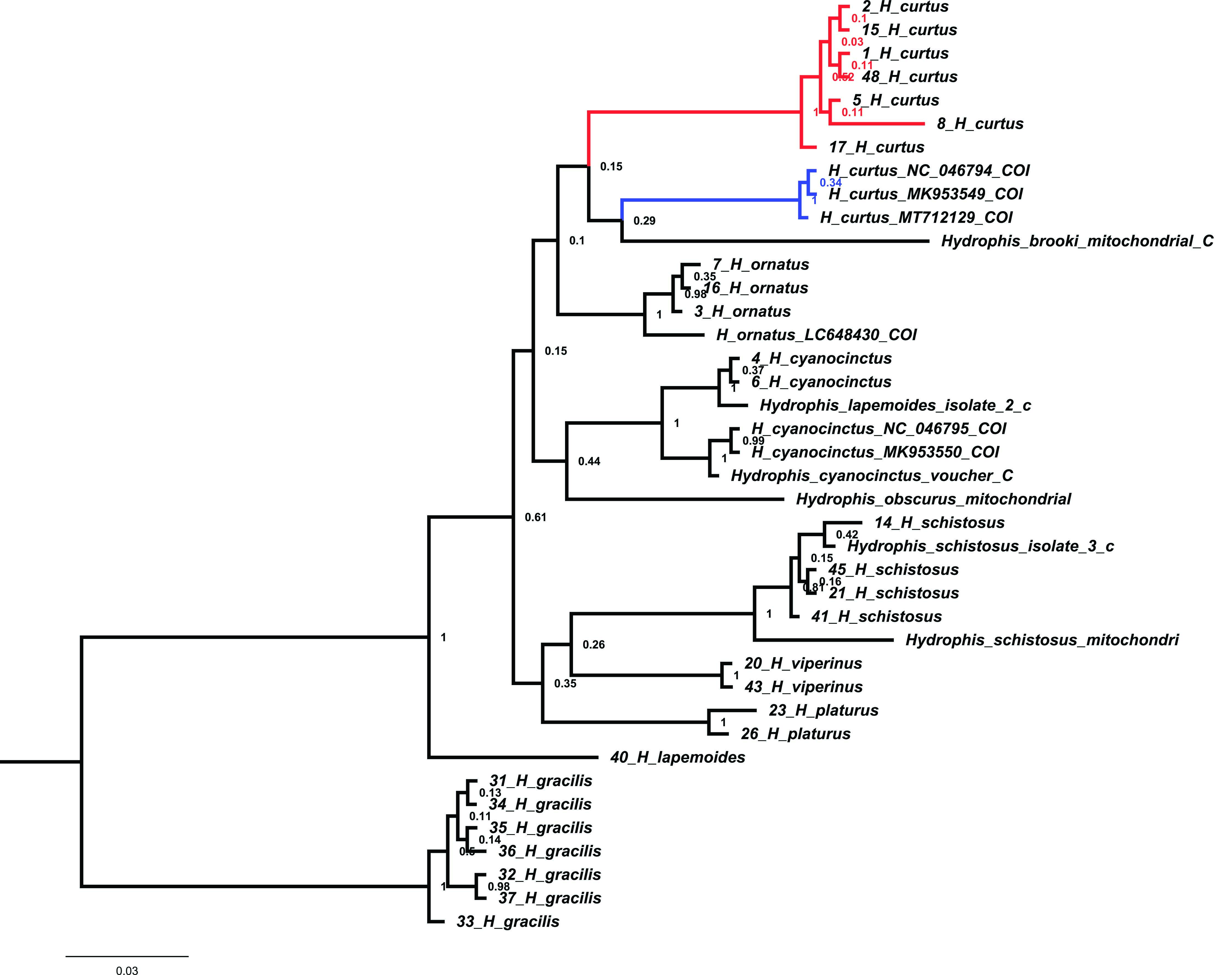

Supplement: Supplementary material 2 — Bayesian tree of COI gene fragment that clearly shows variation in Hydrophiscurtus clade [file zookeys-1158-121_article-101347__-s002.jpg]
